# Toxoplasma gondii AP2XII-2 Contributes to Transcriptional Repression for Sexual Commitment

**DOI:** 10.1128/msphere.00606-22

**Published:** 2023-02-14

**Authors:** Sandeep Srivastava, Michael J. Holmes, Michael W. White, William J. Sullivan

**Affiliations:** a Department of Pharmacology and Toxicology, Indiana University School of Medicine, Indianapolis, Indiana, USA; b Department of Microbiology and Immunology, Indiana University School of Medicine, Indianapolis, Indiana, USA; c Department of Global Health, University of South Florida, Tampa, Florida, USA; University of Georgia

**Keywords:** *Toxoplasma*, apicomplexan parasites, gene expression, sexual development, transcription

## Abstract

Toxoplasma gondii is a widespread protozoan parasite that has a significant impact on human and veterinary health. The parasite undergoes a complex life cycle involving multiple hosts and developmental stages. How *Toxoplasma* transitions between life cycle stages is poorly understood yet central to controlling transmission. Of particular neglect are the factors that contribute to its sexual development, which takes place exclusively in feline intestines. While epigenetic repressors have been shown to play an important role in silencing the spurious gene expression of sexually committed parasites, the specific factors that recruit this generalized machinery to the appropriate genes remain largely unexplored. Here, we establish that a member of the AP2 transcription factor family, AP2XII-2, is targeted to genomic loci associated with sexually committed parasites along with epigenetic regulators of transcriptional silencing, HDAC3 and MORC. Despite its widespread association with gene promoters, AP2XII-2 is required for the silencing of relatively few genes. Using the CUT&Tag (cleavage under targets and tagmentation) methodology, we identify two major genes associated with sexual development downstream of AP2XII-2 control, AP2X-10 and the amino acid hydroxylase AAH1. Our findings show that AP2XII-2 is a key contributor to the gene regulatory pathways modulating *Toxoplasma* sexual development.

**IMPORTANCE** Toxoplasma gondii is a parasite that undergoes its sexual stage exclusively in feline intestines, making cats a major source of transmission. A better understanding of the proteins controlling the parasite’s life cycle stage transitions is needed for the development of new therapies aimed at treating toxoplasmosis and the transmission of the infection. Genes that regulate the sexual stages need to be turned on and off at the appropriate times, activities that are mediated by specific transcription factors that recruit general machinery to silence or activate gene expression. In this study, we identify a transcription factor called AP2XII-2 as being important for the repression of a subset of sexual stage genes, including a sexual stage-specific AP2 factor (AP2X-10) and a protein (AAH1) required to construct the infectious oocysts expelled from infected cats.

## INTRODUCTION

The ubiquitous parasite Toxoplasma gondii (phylum Apicomplexa) infects warm-blooded animals the world over, including approximately one in three people (1). After an initial acute phase of infection by tachyzoites, the parasites redistribute within the solid organs of the infected host and reside as bradyzoites in latent tissue cysts that appear impervious to the immune response and currently available treatments (1). Persistence within the host makes *Toxoplasma* infection an opportunistic infection in those with compromised immune systems (1). Upon immunosuppression, latent bradyzoite stage parasites reconvert into rapidly growing tachyzoites, causing severe tissue destruction that can threaten the life of the infected individual (1).

Given the importance of the interconversion between tachyzoites and bradyzoites for disease progression, the molecular mechanisms governing this stage transition have received a great deal of attention (2). The conversion of tachyzoites to bradyzoites requires the extensive reprogramming of gene expression at multiple levels, directed in part by chromatin-remodeling histone acetyltransferases and deacetylases that are recruited to specific gene loci through their interactions with sequence-specific transcription factors (3, 4). In support of this, members of the AP2 transcription factor family have been found to associate with a cadre of histone-modifying complexes, including GCN5b/ADA2a and HDAC3/MORC (5, 6). AP2 transcription factors harbor a plant-like DNA-binding domain and have been implicated in contributing to the regulation of gene expression in apicomplexan parasites (7).

In contrast, our understanding of the molecular mechanisms governing the rewiring of gene expression during *Toxoplasma* sexual development is far less developed, largely due to a lack of model systems. This is despite evidence showing that the ingestion of the ultimate product of *Toxoplasma* sexual reproduction, sporulated oocysts, accounts for substantial portions of worldwide human infections and mass outbreaks of acute toxoplasmosis (8, 9).

The early stages of *Toxoplasma* sexual development occur in the intestinal lining of cats. Upon ingestion by a feline, the parasites invade intestinal enteroepithelial cells and undergo several rounds of schizogonic replication to produce merozoites (10). Successive rounds of schizogony have been subcategorized into types A to E of the enteroepithelial stage. These presexual stages then undergo gametocytogenesis, ultimately producing motile male microgametes and intracellular female macrogametes. After fertilization, up to a billion immature oocysts are expelled with the cat’s feces (11). Upon exposure to the air, the oocysts sporulate and become capable of transmitting infection (10).

It was recently shown that transcriptional repression mediated by the HDAC3/MORC complex plays an important role in preventing the aberrant expression of genes normally restricted to the sexual stages in felids (6). The HDAC3/MORC complex associates with at least 11 AP2 family transcriptional regulators (6, 12), suggesting that these factors mediate the targeted silencing of felid-specific developmental gene expression, as proposed previously for bradyzoite formation (4).

We initially found that AP2XII-2 associates with AP2IX-4, a transcriptional repressor that helps coordinate bradyzoite formation (12, 13). We then reported that AP2XII-2 is required for the proper progression of parasites through the S phase of the tachyzoite cell cycle and that the knockdown of AP2XII-2 increased the frequency of bradyzoite formation *in vitro* (12). We and others have also reported the association between the HDAC3/MORC complex and AP2XII-2 (6, 12), suggesting that this AP2 family member controls the transcriptional repression of certain genes. However, the specific genes regulated by AP2XII-2 remained unexplored.

To better understand the role of AP2XII-2 in *Toxoplasma* biology, we profiled the genome-wide chromatin-binding sites of AP2XII-2. By adopting the cleavage under targets and tagmentation (CUT&Tag) method for use in *Toxoplasma*, we found a high degree of overlap between AP2XII-2 and HDAC3/MORC target genes. Using a transcriptomics approach, we also found that AP2XII-2 controls the expression of a subset of HDAC3/MORC target genes, including some that are restricted to the sexually committed developmental stages, such as AP2X-10 and the aromatic amino acid hydroxylase AAH1. Our results indicate that AP2XII-2 helps coordinate the recruitment of the HDAC3/MORC complex to specific gene loci to repress developmentally controlled genes.

## RESULTS AND DISCUSSION

### AP2XII-2 shows widespread occupancy at gene promoters and a high degree of overlap with the HDAC3/MORC complex.

**FIG 1:**
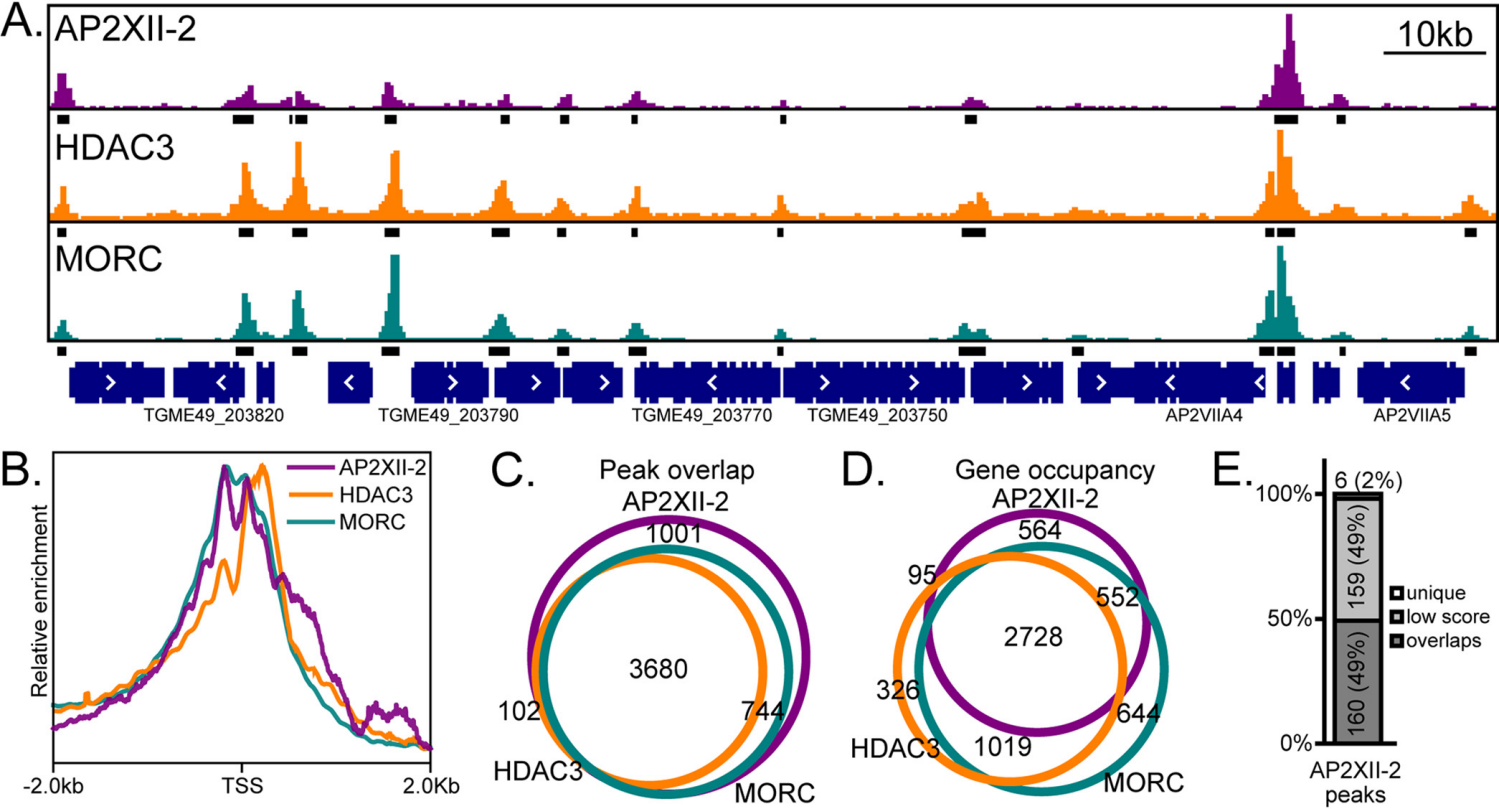
AP2XII-2 genome occupancy significantly overlaps those of HDAC3 and MORC. (A) AP2XII-2 genome occupancy along a segment of chromosome VIIa reveals its association with the promoters of several genes. Statistically enriched segments are shown as black bars directly under AP2XII-2-enriched peaks (purple). Similar distributions and enrichments of HDAC3 and MORC are seen in the orange and teal tracks, respectively. (B) Genome-wide analysis reveals that each member of the transcriptional repression complex associates with the transcriptional start sites (TSS) of genes. (C) Venn diagram indicating that the majority of the AP2XII-2-enriched peaks fall within those identified for HDAC3 and MORC. (D) Venn diagram indicating that the majority of genes occupied by AP2XII-2 are also occupied by HDAC3 and MORC. (E) Analysis of genes flagged as being bound only by AP2XII-2 in panel D. Genes are grouped into subsets of genes whose AP2XII-2 peaks overlap HDAC3/MORC peaks but were affected by gene annotation issues, genes associated with low peak scores, and genes that appear to be uniquely bound by AP2XII-2.

To identify genes that are direct targets of AP2XII-2 in tachyzoites, we applied the CUT&Tag method (14) to profile the genomic loci bound by the factor. To pull down AP2XII-2 for CUT&Tag, we took advantage of a parasite clone that we made previously in the ME49 strain that expresses a hemagglutinin (HA)-tagged version of the endogenous protein (12). The results showed that AP2XII-2-associated regions are widespread across the parasite genome, displaying a total of 5,527 peaks associated with 3,939 genes (Fig. 1A; see also Table S1 in the supplemental material). Consistent with its putative role as a transcriptional regulator, AP2XII-2 is located near the transcriptional start sites of protein-coding genes (Fig. 1B).

10.1128/msphere.00606-22.4 TABLE S1 Chromatin-associated regions of AP2XII-2, HDAC3, and MORC as revealed by CUT&Tag or ChIPseq. Download Table S1, XLSX file, 1.2 MB. Copyright © 2023 Srivastava et al. 2023 Srivastava et al. https://creativecommons.org/licenses/by/4.0/ This content is distributed under the terms of the Creative Commons Attribution 4.0 International license.

Previous reports have demonstrated that AP2XII-2 is a member of the multicomponent HDAC3/MORC complex, which silences genes by inducing heterochromatin formation (6, 12). To corroborate this association, we reanalyzed the existing HDAC3 and MORC chromatin immunoprecipitation sequencing (ChIPseq) data (6) to determine the overlap among HDAC3, MORC, and AP2XII-2 localizations genome-wide (Table S1). Like AP2XII-2, both HDAC3 and MORC are enriched at the transcriptional start sites of protein-coding genes (Fig. 1A and B). Just over 80% of the AP2XII-2-enriched regions overlapped HDAC3- and/or MORC-enriched peaks (Fig. 1C). Additionally, over 85% of the genes associated with AP2XII-2 were also bound by HDAC3 and/or MORC, with a clear plurality of all identified genes bound by the three proteins (Fig. 1D).

In order to determine whether AP2XII-2 is likely to play an HDAC3/MORC-independent role(s), we examined the 564 genes that were flagged as being bound by AP2XII-2 only. After removing rRNA and tRNA genes, we were left with 325 protein-coding genes, most of which were false positives (Fig. 1E). Half of the flagged genes (49%) were due to peak misattributions when genes are arranged in a head-to-head orientation (i.e., likely bidirectional promoters). In this case, the AP2XII-2 peak was assigned to one gene, and the HDAC3 and/or MORC peak was assigned to the other. Gene misattributions due to other reference genome annotation issues, such as overlapping gene models, also fall within this subset. The other half of the genes (49%) were peaks that were called at a low score and may be background or noise upon visual inspection (low-score subset). Of the six remaining genes that are occupied by AP2XII-2 only (unique subset), three showed AP2XII-2 occupancy in areas that also trended toward enrichment for HDAC3/MORC (but did not meet the statistical cutoff). Another gene had an AP2XII-2 peak in the coding DNA sequence (CDS) without any evidence of an alternative transcriptional start site to support an internal cryptic promoter. We cannot exclude the possibility of AP2XII-2 occupying genomic loci in the absence of HDAC3/MORC. However, we find it most likely that all bona fide AP2XII-2-bound sites coincide with HDAC3/MORC occupancy given the rarity of the finding. Given that AP2 domains bind distinct DNA motifs and that HDAC3 and MORC lack DNA-binding domains (15, 16), combined with the previous observation that they operate within a shared complex (6, 12), the high degree of coordination among AP2XII-2-, HDAC3-, and MORC-binding sites strongly suggests that AP2XII-2 is responsible for recruiting the HDAC3/MORC complex to repress specific genes.

### The loss of AP2XII-2 disrupts a small subset of genes in tachyzoites.

**FIG 2:**
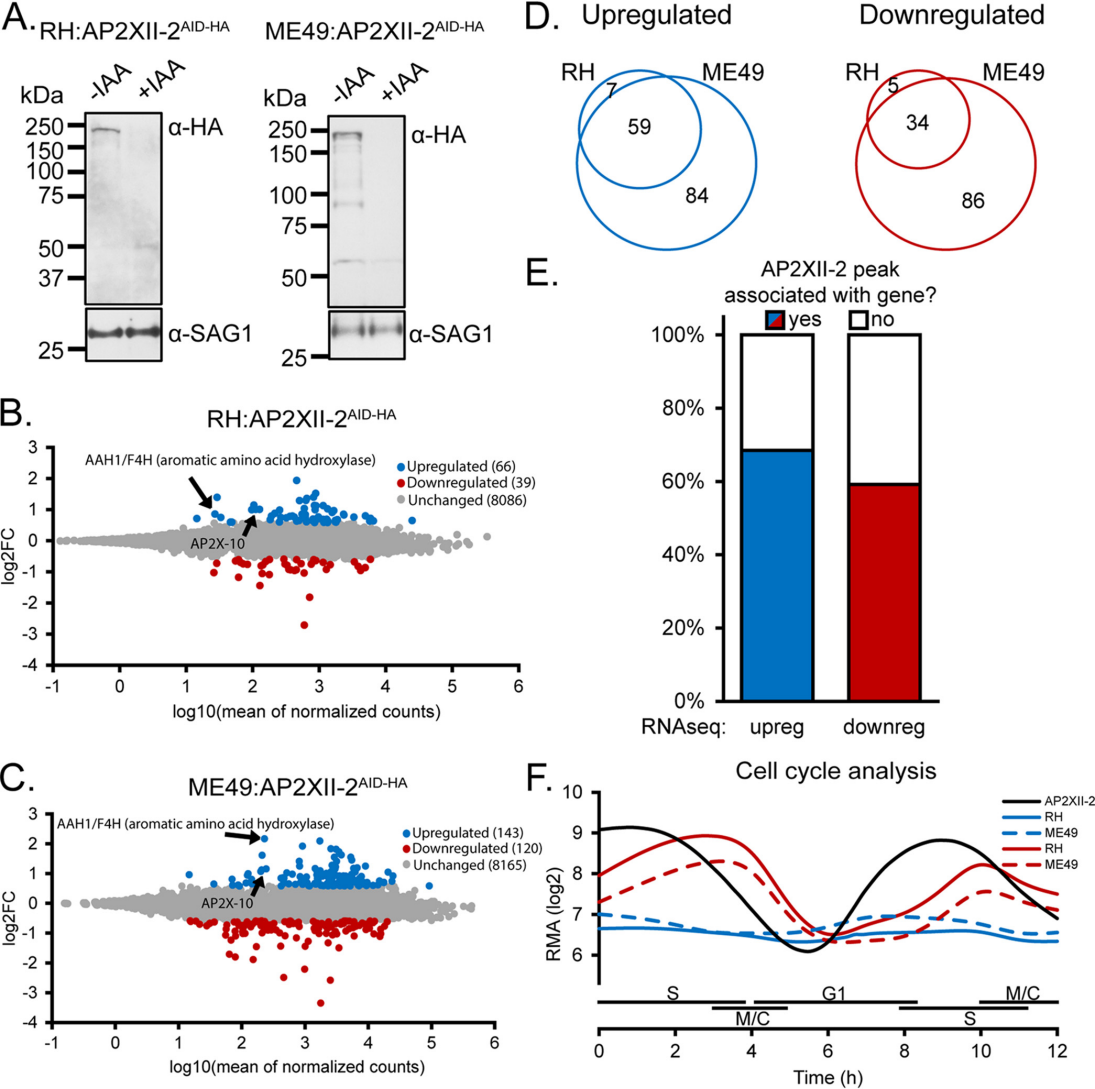
AP2XII-2 depletion leads to relatively little gene dysregulation. (A) Western blotting confirming the IAA-dependent downregulation of AP2XII-2^AID-HA^ protein in RH^TIR1^ or ME49^TIR1^ parasites after 24 h of incubation with 500 μM IAA in culture medium. Blots were probed with anti-SAG1 as a loading control. (B and C) MA plots of differential gene expression of AP2XII-2^AID-HA^ depleted in RH^TIR1^ (B) or ME49^TIR1^ (C) parasites 24 h after IAA addition. Significantly upregulated genes are in blue, and downregulated genes are in red. Genes of interest for further analysis are noted. (D) Venn diagrams showing the overlap of significantly upregulated and downregulated genes in the ME49 and RH genetic backgrounds. (E) Analysis of AP2XII-2 gene occupancy and directionality of dysregulation that occurs in the ME49 strain upon AP2XII-2 depletion. (F) Meta cell cycle expression analysis of significantly upregulated (blue) and downregulated (red) gene sets that respond to AP2XII-2 depletion. The cell cycle profile showing Growth 1 (G1), Synthesis (S), and Mitosis/Cytokinesis (M/C) phases of AP2XII-2 is shown in black as a reference.

Given that AP2XII-2 associates with a large number of gene promoters and likely directs transcriptional silencing through the HDAC3/MORC complex, we wanted to assess the consequences of AP2XII-2 depletion for transcriptional regulation. To maximize the robustness of this experiment, we compared the responses to AP2XII-2 depletion in both type I (RH strain) and type II (ME49 strain) backgrounds using parasites that we made in a previous study (12). These parasites express endogenous AP2XII-2 tagged at its C terminus with a tag comprised of an HA epitope and an auxin-inducible degron (AID) (AP2XII-2^AID-HA^). The AP2XII-2^AID-HA^ protein in both strains was degraded to undetectable levels within 24 h of the addition of 500 μM indole-3-acetic acid (IAA) to the culture medium (Fig. 2A).

Under these conditions, comparative RNA sequencing (RNAseq) analyses revealed that the loss of AP2XII-2 in tachyzoites led to the dysregulation of mRNA levels for a modest number of transcripts in both the RH and ME49 genetic backgrounds (Table S2). In RH parasites lacking AP2XII-2, 66 genes were significantly upregulated and 39 genes were downregulated at least 1.5-fold (Fig. 2B). Similarly, in ME49 parasites, 143 genes were significantly upregulated and 120 genes were downregulated 1.5-fold following the depletion of AP2XII-2 (Fig. 2C). The majority of the dysregulated genes in the RH strain were also dysregulated in the ME49 strain, indicating a high degree of concurrence between strains (Fig. 2D). Surprisingly, in ME49 parasites, there was no correlation between the direction of transcriptional dysregulation and AP2XII-2 gene occupancy as determined by CUT&Tag (Fig. 2E). This observation likely speaks to the complexity of gene regulation in *Toxoplasma*, which can involve multiple AP2 factors and chromatin-remodeling machinery. The depletion of a single factor like AP2XII-2 may not be sufficient to significantly disrupt the expression of most genes in the network. Since AP2XII-2 was also found to interact with AP2IX-4 (12), our observations here may point to a model where AP2 family members cooperatively bind for efficient HDAC3/MORC recruitment to specific gene loci.

10.1128/msphere.00606-22.5 TABLE S2 Differential gene expression analysis resulting from AP2XII-2 depletion in the RH and ME49 backgrounds. Download Table S2, XLSX file, 1.7 MB. Copyright © 2023 Srivastava et al. 2023 Srivastava et al. https://creativecommons.org/licenses/by/4.0/ This content is distributed under the terms of the Creative Commons Attribution 4.0 International license.

Since AP2XII-2 is a cell cycle-regulated factor with expression peaking during the S/M phase (12, 17), we assessed whether the dysregulated genes were also subject to cyclic control. Using the cell cycle microarray data available at ToxoDB.org (17, 18), we determined the average cell cycle profiles for the genes that were up- or downregulated in response to AP2XII-2 depletion and compared them to the cell cycle pattern exhibited by AP2XII-2 (Fig. 2F). Downregulated genes tend to be cell cycle regulated, with a valley of expression that spans mid-G_1_ phase, in line with our previous observation that AP2XII-2 depletion delays the cell cycle after centrosome duplication (12). The expanded G_1_-phase occupancy induced by AP2XII-2 depletion may point to a link between the transcriptional repression of AP2XII-2 target genes and the cell cycle. Despite not displaying cell cycle regulation, the upregulated genes were expressed at lower robust multichip average (RMA) expression values. The relatively low abundance of these genes in tachyzoites could indicate that they are normally subject to transcriptional repression. Given the association of AP2XII-2 with the HDAC3/MORC transcriptional repression complex (6, 12), we suspected that these upregulated genes could be subject to regulation by AP2XII-2 via the recruitment of HDAC3/MORC.

### The loss of AP2XII-2 increases the expression of genes normally restricted to latent and sexually committed parasites.

Since the HDAC3/MORC complex has been reported to regulate the expression of genes relevant for the bradyzoite and sexually committed stages (6), we first determined whether the genes bound by AP2XII-2 were enriched for these stages. Using data available at ToxoDB.org (18, 19), we determined the stage at which each gene achieves maximal expression as measured by RNA abundance. AP2XII-2 occupancy did not enrich for genes associated with any stage above their representation in the total genomic content; each developmental stage was well represented (Fig. S1).

10.1128/msphere.00606-22.1 FIG S1 Stage specificity evaluation of genes bound by AP2XII-2. Breakdown of stages at which each gene shows maximal transcript abundance for genes bound by AP2XII-2 (A) or for all protein-coding genes (B). Download FIG S1, TIF file, 0.4 MB. Copyright © 2023 Srivastava et al. 2023 Srivastava et al. https://creativecommons.org/licenses/by/4.0/ This content is distributed under the terms of the Creative Commons Attribution 4.0 International license.

In an alternative approach, to identify genes that are most likely to be direct targets of the AP2XII-2-directed HDAC3/MORC complex, we looked for genes that were upregulated in either ME49 or RH strain parasites (i) upon AP2XII-2 depletion, (ii) after 24 h of MORC depletion (Fig. S2A), and (iii) after 18 h of incubation with the HDAC3 inhibitor FR235222 (Fig. S2B). The latter two criteria were assessed using publicly available data and were also generated from a type II parasite strain (Table S3) (6). In addition, we verified that each gene’s promoter was occupied by AP2XII-2, HDAC3, and MORC. Five genes met these stringent filtering criteria (Table 1).

**TABLE 1:** Genes occupied by AP2XII-2, HDAC3, and MORC that are consistently upregulated upon their depletion or inhibition^*a*^

Gene ID	Product description	Log_2_FC value			
		AP2XII-2 depletion		MORC	HDAC3
		ME49	RH		
TGME49_212710	F4H	2.18	0.86	4.29	3.74
TGME49_287510	Aromatic amino acid hydrolase (AAH1)	0.58	1.40	4.91	3.22
TGME49_318750	TgDPA	1.82	1.30	1.39	1.40
TGME49_215340	AP2X-10 (fragment)	1.16	1.01	5.19	3.57
TGME49_215343	Hypothetical protein (AP2X-10 fragment)	1.13	1.00	5.27	3.45

a Log_2_FC, log_2_ fold change; F4H, phenylalanine-4-hydroxylase; TgDPA, T. gondii deoxyribose-phosphate aldolase.

10.1128/msphere.00606-22.2 FIG S2 Reanalysis of transcriptomic changes that occur upon the impairment of the HDAC3/MORC complex. Differential gene expression analysis was performed for HDAC3 inhibition (A) and IAA-induced MORC depletion (B), each conducted as it was for newly generated AP2XII-2 depletion data. Download FIG S2, TIF file, 0.8 MB. Copyright © 2023 Srivastava et al. 2023 Srivastava et al. https://creativecommons.org/licenses/by/4.0/ This content is distributed under the terms of the Creative Commons Attribution 4.0 International license.

10.1128/msphere.00606-22.6 TABLE S3 Differential gene expression analysis resulting from HDAC3 inhibition or MORC depletion. Download Table S3, XLSX file, 1.5 MB. Copyright © 2023 Srivastava et al. 2023 Srivastava et al. https://creativecommons.org/licenses/by/4.0/ This content is distributed under the terms of the Creative Commons Attribution 4.0 International license.

The auxin-induced depletion of MORC and inhibition of HDAC3 in tachyzoites induced the upregulation of bradyzoite-specific genes as well as genes associated with sexual stages (6, 20). Notably, when assessing the degree of dysregulation for the genes outlined in Table 1, the effect was more pronounced for MORC and HDAC3 than for AP2XII-2 depletion. This observation is consistent with a model whereby multiple AP2 family members cooperatively recruit the HDAC3/MORC complex to genomic loci.

It has been proposed that parasites must pass through the bradyzoite stage before initiating sexual commitment (6, 21). Our analysis (Table 1) identified a single bradyzoite-specific gene, T. gondii deoxyribose-phosphate aldolase (TgDPA) (22), to be reliably upregulated under all three treatments that were assessed, strongly suggesting that its expression is controlled by an AP2XII-2-directed HDAC3/MORC complex. The roles that TgDPA plays in bradyzoite biology and the parasite’s potential commitment to sexual development remain to be elucidated.

Interestingly, the AP2 family member AP2X-10, whose current fragmented gene annotation incorrectly assigns the gene to both TGME49_215340 and TGME49_215343, is upregulated when either AP2XII-2, MORC, or HDAC3 is impaired, independent of the parasite strain (Table 1). AP2X-10 expression has been noted to normally be restricted to the enteroepithelial and oocyst stages; it is the most abundant of all AP2 factors in oocysts, being expressed at the 96th percentile (19, 23, 24). Seventeen AP2 family members have been proposed to act downstream of the HDAC3/MORC complex to regulate various developmental trajectories, with AP2X-10 being predicted to guide micro- and/or macrogamete development (6). The results of our present study are consistent with AP2XII-2 coordinating the repression of AP2X-10, placing the former above AP2X-10 in a transcriptional regulatory cascade that contributes to the coordination of *Toxoplasma* sexual development. Future studies to determine the genes directly controlled by AP2X-10 and clarify its role in sexual development are warranted.

### AAH family gene organization.

Two aromatic amino acid hydroxylase enzymes, AAH1 and phenylalanine-4-hydroxylase (F4H), not only are counted among the highest-upregulated genes upon AP2XII-2, MORC, and HDAC3 inhibition (Table 1) but also have been reported to be important for oocyst maturation (25). Interestingly, we found that F4H was upregulated in the ME49 but not the RH strain, while AAH1 showed the opposite trend (Table 1). These observations prompted us to further examine the entire aromatic amino acid hydroxylase family in *Toxoplasma*, which consists of three annotated members, TGME49_287510 (AAH1), TGME49_212740 (AAH2), and TGME49_212710 (F4H).

**FIG 3:**
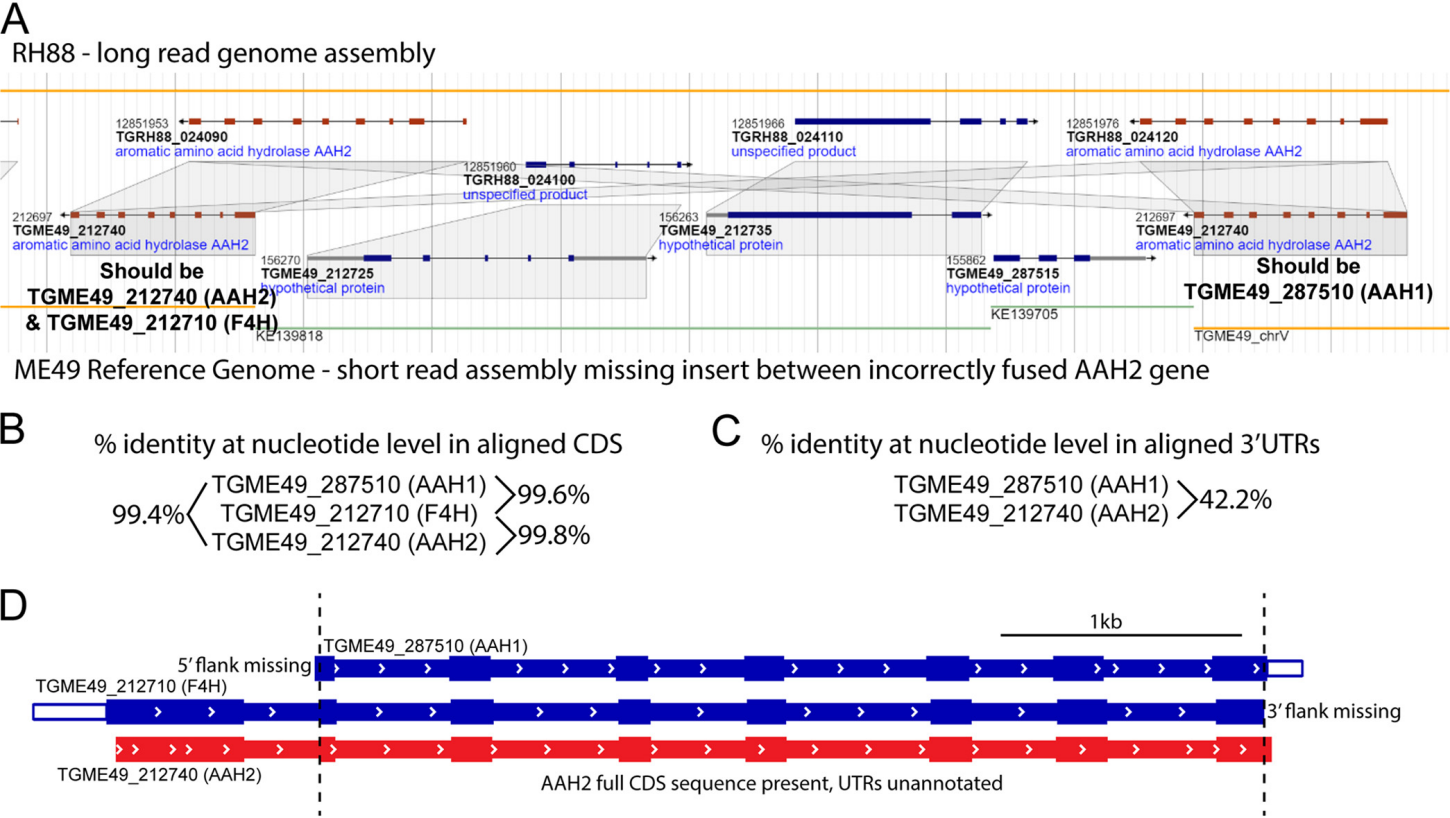
Disentanglement of the aromatic amino acid hydroxylase family of genes on chromosome V. (A) Genomic alignment and syntenic analysis of RH88 and ME49 genomes available at ToxoDB.org reveal the correct positioning of the unplaced contigs under GenBank accession numbers KE139705 and KE139818 into chromosome V, which was missed due to the incorrect fusion of the AAH1/2 genes in the ME49 reference strain. Suggested changes to the annotations of AAH1 and AAH2 are based on sequence alignments of the 3′-flanking regions in both genes across both parasite strains. (B) Multiple pairwise alignments of CDS regions that are present in each AAH gene fragment reveal a high degree of identity at the nucleotide level, which obfuscates gene calling during differential gene expression analysis. (C) Pairwise alignment of the 3′-flanking regions reveals a clear way to discriminate both the AAH1 and AAH2 genes for C-terminal tagging at endogenous loci. (D) Outline of AAH family gene fragments available in the ME49 reference strain. Missing fragments at either the 5′ or the 3′ terminus are indicated. Conserved CDS regions that were used for multiple-sequence alignment are delimited by dotted lines.

Previous reports have indicated that the current ME49 genome assembly available at ToxoDB.org has incorrectly fused a tandem repeat of the AAH2 gene (25, 26). Before proceeding with our analysis, we had to ensure the fidelity of the AAH2 gene sequence. To that end, we compared the ME49 genome assembly with the RH88 assembly since the former was generated using short-read sequencing technology, which can have difficulty with repetitive regions (Fig. 3A). The RH88 genome was constructed based on long-read sequencing technology, which is better at determining the overall genome architecture (27). This comparison revealed that, indeed, two members of the AAH family should appear on chromosome V of the ME49 genome, spaced apart by the unplaced contigs under GenBank accession numbers KE139705 and KE139818 (Fig. 3A).

The three ME49 members of the AAH family share over 99.4% identity at the nucleotide level in their coding sequences (Fig. 3B), suggesting that the vast majority of RNAseq reads mapping to the CDS region would have been assigned to all AAH genes indiscriminately; this spurious read assignment may indicate that putative strain-specific upregulation of AAH1 or F4H could occur by chance (Table 1). Given the high degree of sequence identity between AAH family members, we examined their flanking sequences to differentiate them. AAH1 and AAH2 have unique 3′-end-flanking sequences (Fig. 3C); however, the unplaced contig under GenBank accession number KE139818 abruptly ends, leaving the annotated F4H gene (TGME49_212710) without a 3′-untranslated region (UTR) sequence (Fig. 3D). Analysis of the unique regions flanking the 5′ and 3′ ends of the AAH family by sequence alignment comparison with the RH88 genome architecture allowed us to place AAH1 and AAH2 onto chromosome V and determine that F4H and AAH2 are likely the same gene (Fig. 3A). However, it should be noted that a previously conducted copy number variant analysis of the AAH family in *Toxoplasma* indicated that ME49 parasites harbor three copies of AAH genes, whereas type I parasites carry only two (25). We were unable to map the location of the additional AAH family member with our strategy of comparing ME49 and RH genome architectures.

### Depletion of AP2XII-2 derepresses AAH1 but not AAH2.

**FIG 4:**
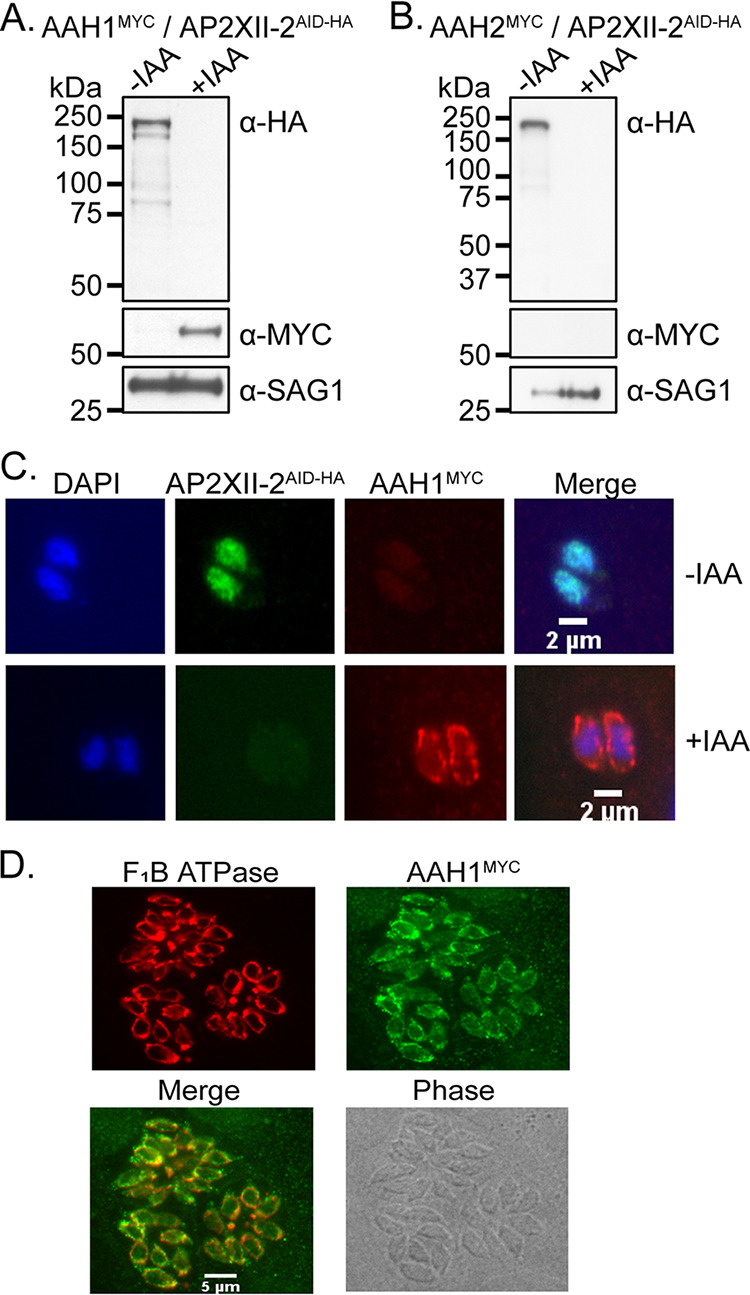
Depletion of AP2XII-2 causes derepression of AAH1 gene expression. (A and B) Western blotting demonstrating that AAH1 (A) and not AAH2 (B) is expressed upon IAA-induced depletion of AP2XII-2. Both genes were endogenously tagged at their C termini with MYC epitope tags. Blots were probed with anti-SAG1 as a loading control. (C) AAH1 expression is observed in a concentric pattern around the nucleus upon AP2XII-2 depletion. (D) AAH1 is expressed in the vicinity of the parasite mitochondrial marker F_1_B ATPase.

To distinguish which AAH family member(s) is upregulated upon AP2XII-2 depletion, we endogenously tagged AAH1 and AAH2 with MYC epitopes at their C termini in ME49 AP2XII-2^AID-HA^ parasites. The tagging of these genes was confirmed by verifying successful recombination at each genetic locus by PCR analysis (Fig. S3). In untreated parasites, AAH1^MYC^ was not detectable by Western blotting, whereas AAH1^MYC^ appeared at the expected size of 62 kDa upon the IAA-induced loss of AP2XII-2 (Fig. 4A). The expression of AAH2^MYC^ was not seen under either condition (Fig. 4B).

10.1128/msphere.00606-22.3 FIG S3 Validation of endogenous tagging of AAH1 and AAH2. (A) AAH1 was endogenously tagged with a MYC epitope tag using a CAS9-assisted approach to facilitate double homologous recombination, as indicated. (B) AAH2 was endogenously tagged with a MYC epitope tag using a single-crossover approach, as indicated. (C and D) Diagnostic PCRs checking for the correct integration (I) or the unmodified endogenous locus (E) were performed on parental and tagged lines. Specific regions selected for PCR analysis are highlighted in panels A and B. Download FIG S3, TIF file, 2.1 MB. Copyright © 2023 Srivastava et al. 2023 Srivastava et al. https://creativecommons.org/licenses/by/4.0/ This content is distributed under the terms of the Creative Commons Attribution 4.0 International license.

Neither AAH family member is typically expressed in asexual parasites (26), suggesting that they are maintained in a transcriptionally repressed state in non-sexually committed parasites. Our results suggest that AAH genes may be regulated by different HDAC3/MORC-AP2 complexes, with AP2XII-2 directing the silencing of AAH1 but not AAH2. However, we cannot rule out the possibility that AAH1 and AAH2 have a parasite strain-specific dependence on AP2XII-2. Since parasites lacking AAH1 have severe defects in infection of the cat intestine and oocyst production (25), we propose that AP2XII-2 is a key factor required for the completion of *Toxoplasma* sexual reproduction.

We next examined AAH1 expression by an immunofluorescence assay (IFA) and found that AAH1^MYC^ was localized around the nuclei of tachyzoites only upon AP2XII-2 depletion (Fig. 4C). This staining pattern is reminiscent of the *Toxoplasma* mitochondrion. To further characterize the AAH1^MYC^ localization, we costained AP2XII-2^AID-HA^ knockdown parasites with antibodies to the F_1_B ATPase mitochondrial marker (28) and the MYC epitope tag, which confirmed the colocalization of AAH1^MYC^ with the mitochondrion in tachyzoites (Fig. 4D).

The AAH family of enzymes is responsible for converting phenylalanine to tyrosine and tyrosine to 3,4-dihydroxy-l-phenylalanine (l-DOPA) (29). The latter compound is a precursor for the formation of dityrosine protein cross-links that are thought to play critical structural roles in *Toxoplasma* oocyst wall integrity and formation (25). Given the role that AAH family enzymes play in ensuring oocyst development and wall integrity, we were surprised to see AAH1 localized to the parasite mitochondrion. Interestingly, a previous report demonstrated that AAH2 overexpression in bradyzoites gave rise to a staining pattern consistent with a mitochondrial localization (26), suggesting that the mitochondrion might act as a hub for AAH enzymatic activity. Future studies using bona fide sexually committed parasites are needed to verify this possibility.

### Conclusion.

We have determined that in addition to affecting tachyzoite cell cycle progression (12), AP2XII-2 also plays a role in coordinating *Toxoplasma* sexual development. This may indicate that AP2XII-2 plays different roles in different developmental stages or may suggest that the commitment to sexual development is a cell cycle-dependent phenomenon, as has been demonstrated for the tachyzoite-to-bradyzoite transition (7, 30). Consistent with this model, either HDAC3 inhibition or MORC depletion also stalls cell cycle progression and leads to increased bradyzoite and sexual stage gene expression (6, 20) albeit to a greater extent than does AP2XII-2 depletion. Given the association of AP2XII-2 with AP2IX-4 (12) and the observation that AP2XII-2 depletion appears to cause a milder version of the HDAC3 and MORC phenotypes, we propose that AP2XII-2 is likely one of several AP2 family members that bind cooperatively to recruit the HDAC3/MORC complex to specific genomic loci. Since the HDAC3/MORC complex has been reported to interact with 11 AP2 family members, it is most likely that HDAC3/MORC complexes display heterogeneity in AP2 family member composition. While some AP2 factors may work cooperatively with AP2XII-2, others are likely mutually exclusive.

On the other hand, a number of reports demonstrate that *Toxoplasma* studies investigating gene regulation using *in vitro* culture systems lack the fidelity of *in vivo* parasite development (31–33). This is particularly relevant when examining the roles of AP2 family members in gene regulation (33). Notably, a previous report indicating that AP2IX-9 is a repressor of bradyzoite formation was later contradicted using developmentally competent *Toxoplasma* models (15, 23, 33). Given that we have reported that AP2XII-2 impacts both cell cycle regulation (12) and the expression of sexually committed genes, we cannot rule out the possibility that misexpression of AP2XII-2 may have resulted in “transcriptional confusion” that impacted tachyzoite cell cycle dynamics. Examining the role that AP2XII-2 plays throughout the complete life cycle stages of *Toxoplasma* development is warranted.

The intrinsic factors responsible for regulating *Toxoplasma* sexual development have been understudied due to the lack of amenable systems. This is beginning to change with the pivotal discoveries of the upstream extrinsic signals required to initiate sexual development and the requirement for widespread transcriptional repression to coordinate the process (6, 34). The precise contribution and role of AP2XII-2 in the sexual development process will require evaluation in such models. Additional future work will undoubtedly focus on disentangling other gene regulatory networks responsible for initiating and managing *Toxoplasma* sexual development. Here, we have begun to unravel one of the networks responsible for this process by placing the AP2XII-2-directed HDAC3/MORC complex above two genes that function in sexual stages, AP2X-10 and AAH1. In addition to utilizing a candidate-based approach, as we have relied on for this study, a recent report highlighting inhibitors of AP2 family member activity may provide yet another tool for interrogating the role of AP2 factors in *Toxoplasma* developmental transitions (35).

## MATERIALS AND METHODS

### Parasite culture.

Human foreskin fibroblast (HFF) cells (ATCC SCRC-104) were maintained in Dulbecco’s modified Eagle medium (DMEM; Corning) supplemented with 10% fetal bovine serum (FBS; R&D Systems), 100 μg/mL streptomycin, and 100 U/mL penicillin, as described previously (36). RH and ME49 parasites were cultured in DMEM supplemented with 1% and 5% heat-inactivated FBS, respectively, 100 μg/mL streptomycin, and 100 U/mL penicillin. Parasites were maintained in a humidified incubator at 37°C with 5% CO_2_. Transfected and clonal strains were maintained in selection medium supplemented with 1 μM pyrimethamine or a combination of 25 μg mycophenolic acid and 50 μg xanthine as appropriate. The control of AP2XII-2 expression was achieved by the addition of either 500 μM indole-3-acetic acid or an equivalent amount of an ethanol vehicle to the culture medium for 24 h before analysis.

### Generation of endogenously tagged AAH1^MYC^ or AAH2^MYC^.

The ME49-TIR1:AP2XII-2^AID-HA^ strain that we generated in a previous study (12) was used as the parental strain for generating dually tagged AAH1^MYC^ and AAH2^MYC^ parasites. For AAH1, the guide RNA sequence from the pSAG1::CAS9-U6::sgUPRT plasmid (Addgene plasmid 54467) (37) was mutagenized to target the stop codon of AAH1. All primers and sequences are listed in Table S4 in the supplemental material. A donor repair template with regions of homology to the AAH1 CDS and 3′ UTR was made to incorporate the MYC tag and the hypoxanthine-xanthine-guanine phosphoribosyl transferase (HXGPRT) selection cassette. Parasites were transfected with 25 μg of a CAS9 plasmid and 25 μg of the donor repair template and cultured with 25 μg of mycophenolic acid and 50 μg of xanthine for 7 to 8 days, and single clones were isolated by limiting dilution in 96-well plates. The isolated single clones were confirmed for C-terminal tagging by PCR of their genomic DNA (Fig. S3).

10.1128/msphere.00606-22.7 TABLE S4 List of primers used in this study. Download Table S4, XLSX file, 0.01 MB. Copyright © 2023 Srivastava et al. 2023 Srivastava et al. https://creativecommons.org/licenses/by/4.0/ This content is distributed under the terms of the Creative Commons Attribution 4.0 International license.

To generate AAH2^MYC^ parasites, specific primers were used to clone ~2 kb of the AAH2 gene into the PacI site of the pLIC-3×MYC-HXGPRT plasmid. Fifty micrograms of the final construct was linearized with the NheI restriction endonuclease and transfected into the ME49-TIR1:AP2XII-2^AID-HA^ strain. Transgenic parasites were selected for 7 to 8 days under drug pressure and subjected to limiting dilution in 96-well plates. Single clones were confirmed for the integration of the MYC tag at the C terminus (Fig. S3).

### Western blotting.

Parasites growing in confluent HFF cells were mechanically lysed and filtered to remove host cell debris, followed by centrifugation. Parasite pellets were lysed in NuPAGE buffer, sonicated, and boiled for 5 min. Parasite lysates were centrifuged briefly to remove debris and separated on 4 to 12% NuPAGE gels (Invitrogen). Separated proteins were transferred to a polyvinylidene difluoride (PVDF) membrane and blocked in a solution containing 5% nonfat dry milk and Tris-buffered saline–Tween (TBST) for 1 h at room temperature. The blocked PVDF membranes were incubated with anti-HA (1:2,000) (Roche), anti-MYC (1:3,000) (Cell Signaling), or anti-SAG1 (1:5,000) (Thermo Fisher) antibodies in blocking buffer at 4°C overnight with gentle shaking. Membranes were washed with TBST and incubated with horseradish peroxidase (HRP)-conjugated anti-rat or anti-mouse antibody (1:2,000) (GE Healthcare) for 1 h at room temperature. After washing with TBST, membranes were developed with the SuperSignal West Femto substrate (Thermo Fisher).

### Immunofluorescence assay.

Parasites growing in HFF monolayers were fixed in a 4% paraformaldehyde solution for 15 min at room temperature, followed by washing with phosphate-buffered saline (PBS). Fixed cells were permeabilized and blocked with blocking buffer (3% bovine serum albumin [BSA] plus 0.2% Triton X-100 in PBS) for 1 h. Cells were incubated with primary antibodies in blocking buffer at 4°C overnight. Cells were washed with PBS and finally incubated with 4′,6-diamidino-2-phenylindole (DAPI) (1:1,000) and Alexa Fluor secondary antibodies coupled with the desired fluorophore for 1 h at room temperature. Cells were washed and mounted with Prolong gold antifade reagent (Invitrogen) before analysis with a Leica DMI6000 B inverted microscope. The following antibodies were used for IFAs: rabbit anti-HA (1:2,000) (catalog number H6908; Sigma), mouse anti-MYC monoclonal antibody (mAb) (1:1000) (clone 9B11; Cell Signaling), rabbit anti-MYC (1:2,000) (catalog number PA1-981; Thermo Fisher), and mouse mAb 5F4 (1:5,000) (F_1_B ATPase, a gift from P. Bradley). Alexa Fluor 488/594 secondary antibodies (1:2,000) (Thermo Fisher) were used as appropriate.

### RNA sequencing.

Parasites were allowed to invade confluent HFF monolayers for 24 h. Infected HFF monolayers were scraped, syringe lysed with a 23-gauge needle to release intracellular parasites, and passed through a 3-μm filter. Filtered parasites were centrifuged at 300 × *g* for 15 min, and DMEM was removed to isolate the parasite pellet. The parasite pellet was resuspended in 1× PBS and centrifuged at 300 × *g* for 15 min, twice. Total RNA was extracted with TRIzol reagent (Invitrogen) according to the manufacturer’s protocol and measured using a Nanodrop spectrophotometer. RNAseq libraries were prepared from poly(A)-purified mRNA by Azenta using standard Illumina protocols. Libraries were sequenced with 150-bp paired-end (PE) reads.

### RNAseq data analysis.

RNAseq read data generated from either the RH or the ME49 genetic background were aligned to the ME49 reference sequence (v52) with hisat2 using default settings (38). Differential gene expression analysis was conducted with DESeq2 using default settings (39). Data pertaining to HDAC3 and MORC impairment (6) were obtained from the GEO under accession number GSE136123.

### CUT&Tag assay.

Analysis of chromatin occupancy for ME49^TIR1^ parasites expressing AP2XII-2^AID-HA^ was performed by CUT&Tag profiling (14). Briefly, 2 × 10^7^ intracellular tachyzoites were syringe lysed at 24 h postinvasion to release fresh parasites. Parasites were centrifuged at 600 × *g* for 10 min at room temperature. Parasite pellets were then processed according to the manufacturer’s protocol (CUT&Tag-IT assay kit, anti-rabbit, catalog number 53160; Active Motif). Libraries were sequenced by Azenta with PE 2× 150-bp reads.

### Chromatin occupancy data analysis.

Sequencing data were aligned to the ME49 genome (v52) with bowtie2 (40), allowing for mate dovetailing. Peaks were called with MACS2 using default settings (41). Peak annotation was performed with Homer using a 2-kb cutoff distance (42). Peak overlap analysis was conducted with the bedtools overlap function (43). Raw data pertaining to ChIPseq of MORC and HDAC3 (6) were obtained from the GEO under accession number GSE136060.

### Data availability.

Raw sequencing data and processed files generated in this study are available at the GEO repository under accession number GSE217226 for RNAseq analysis, and under accession number GSE217220 for AP2XII-2 CUT&Tag.
