# Supplementary figures and images for: Toxoplasma gondii AP2XII-2 Contributes to Transcriptional Repression for Sexual Commitment
Source: mSphere. 2023 Feb 14;8(2):e00606-22. doi: 10.1128/msphere.00606-22 (PMC10117075; doi:10.1128/msphere.00606-22)

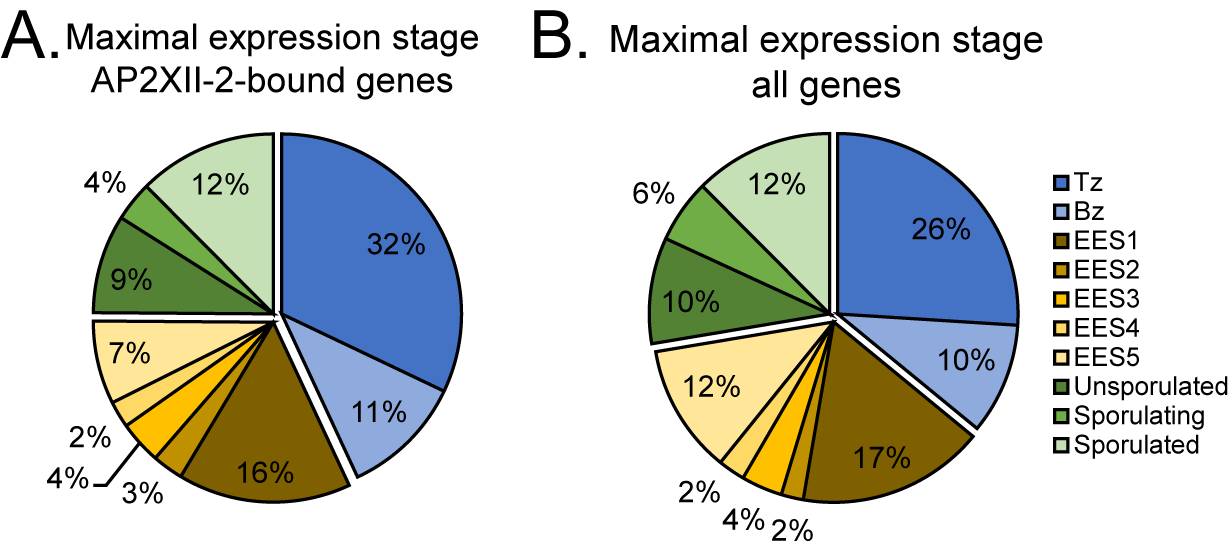

Supplement: FIG S1 [file msphere.00606-22-s0001.tif]

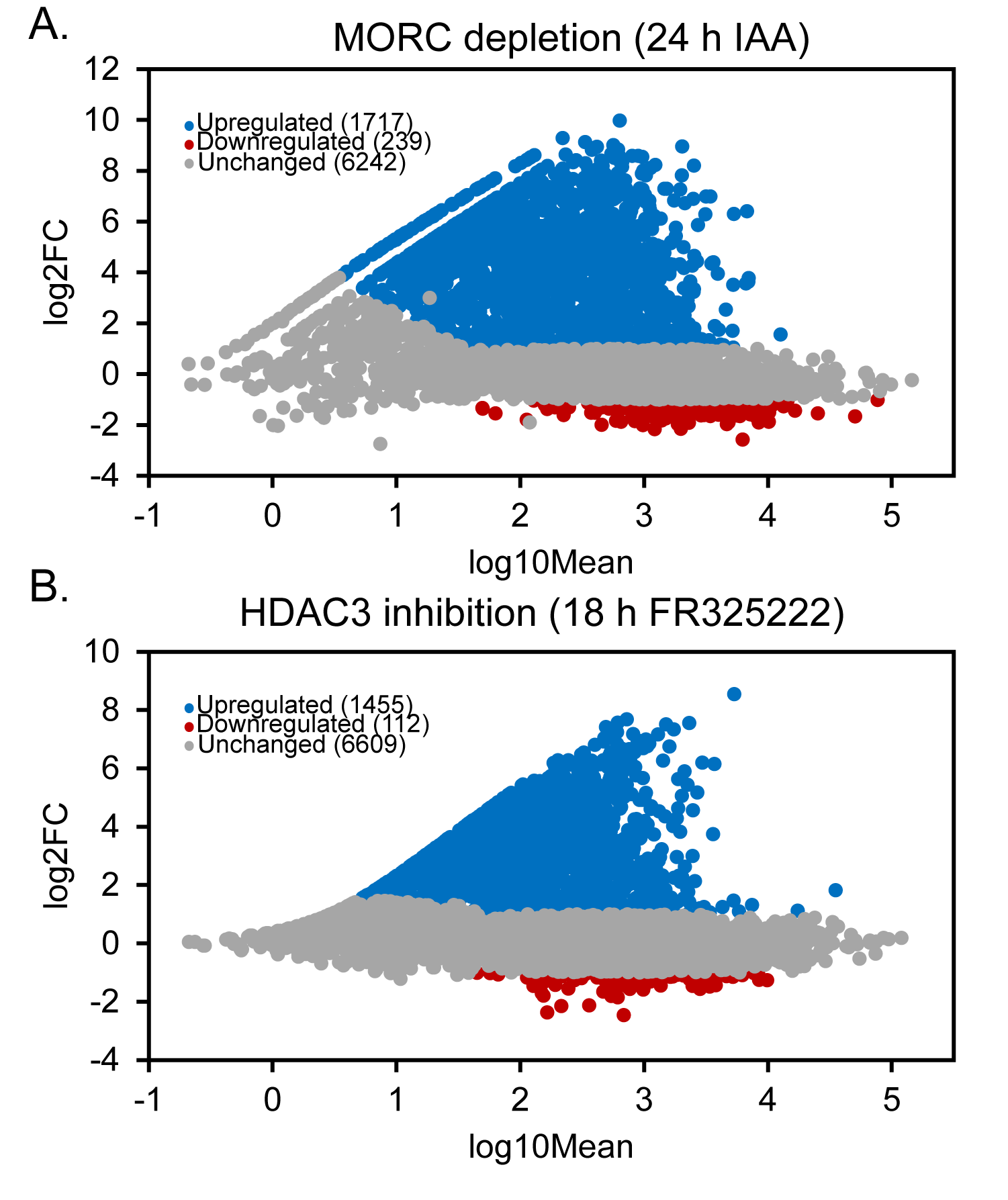

Supplement: FIG S2 [file msphere.00606-22-s0002.tif]

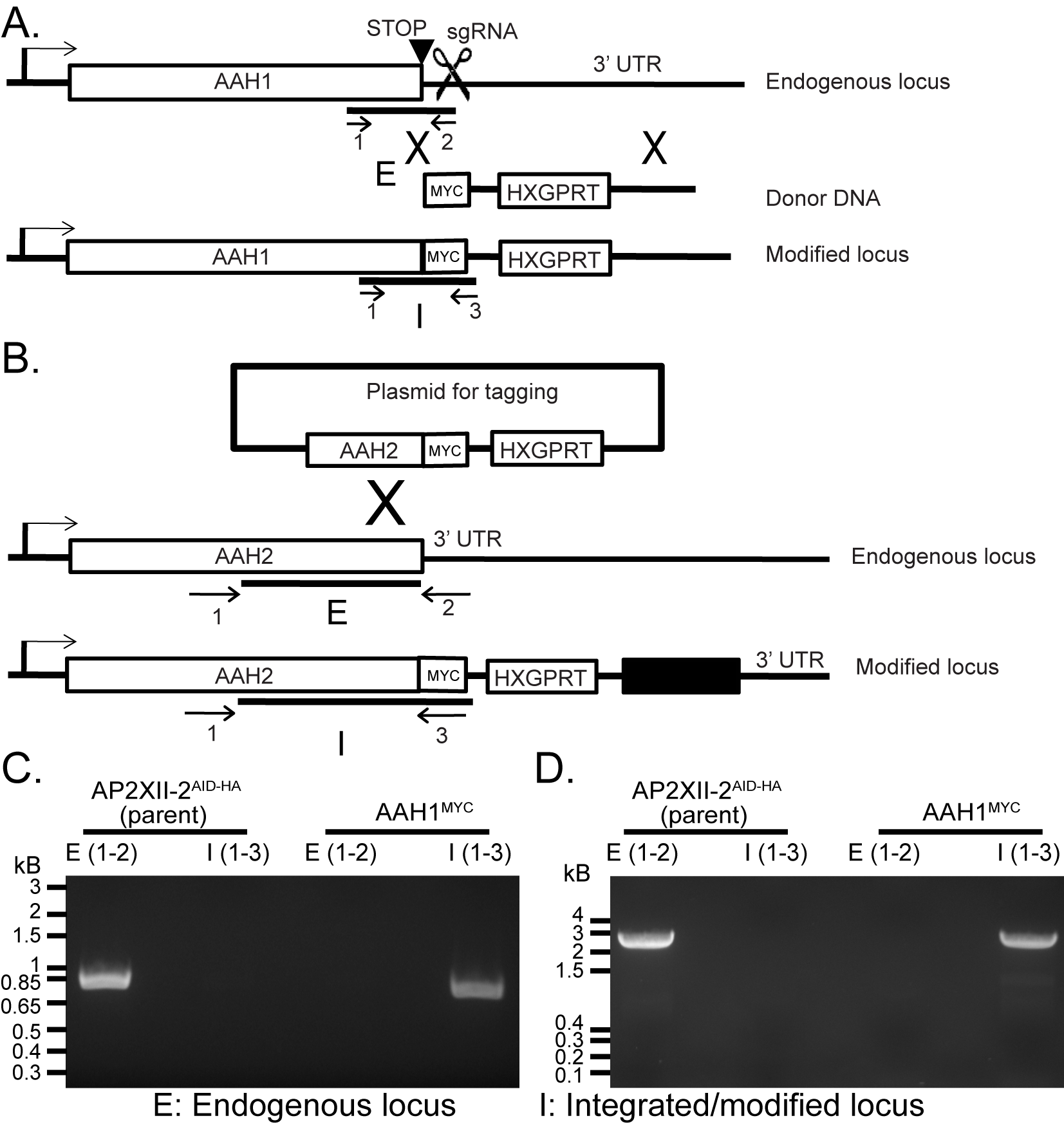

Supplement: FIG S3 [file msphere.00606-22-s0003.tif]
